# A novel *UBAP1* truncated variant in a Chinese family with hereditary spastic paraplegia

**DOI:** 10.1002/mgg3.1927

**Published:** 2022-03-29

**Authors:** Qiao Wei, Pei‐Shan Wang, Hai‐Lin Dong, Wen‐Jiao Luo, Zhi‐Ying Wu, Hong‐Fu Li

**Affiliations:** ^1^ Department of Neurology and Research Center of Neurology Second Affiliated Hospital, Zhejiang University School of Medicine Hangzhou China; ^2^ Key Laboratory of Medical Neurobiology of Zhejiang Province Second Affiliated Hospital, Zhejiang University School of Medicine Hangzhou China

**Keywords:** Chinese, hereditary spastic paraplegias, *UBAP1*

## Abstract

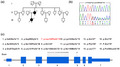

To the editor,

Hereditary spastic paraplegias (HSPs) are a group of neurodegenerative diseases predominately presented with weakness and spasticity in lower extremities. HSPs have high clinical and genetic heterogeneity and over 80 genes or loci have been linked to HSP over the past two decades (Mackay‐Sim, 2021). Even so, appropriately 50% of affected individuals were still not genetically diagnosed. In 2019, two studies (Farazi Fard et al., 2019; Lin et al., 2019) identified pathological truncating variants within *UBAP1* in autosomal dominant HSP pedigrees. These families are from Iran, USA, Germany, Canada, Bulgaria, Spain, and China, respectively, implying the diverse geographic origin for the *UBAP1* variants. The phenotypes are predominantly pure early‐onset HSP in these families (MIM # 618418). In this study, we reported a novel *UBAP1* (NM_016525.5) truncating variant c.371dupT (p.Leu124Phefs*15) in a Chinese autosomal dominant HSP pedigree (Figure 1a). This study was approved by the Ethics Committee of Second Affiliated Hospital, Zhejiang University School of Medicine and written informed consents were obtained from the participants.

**FIGURE 1:**
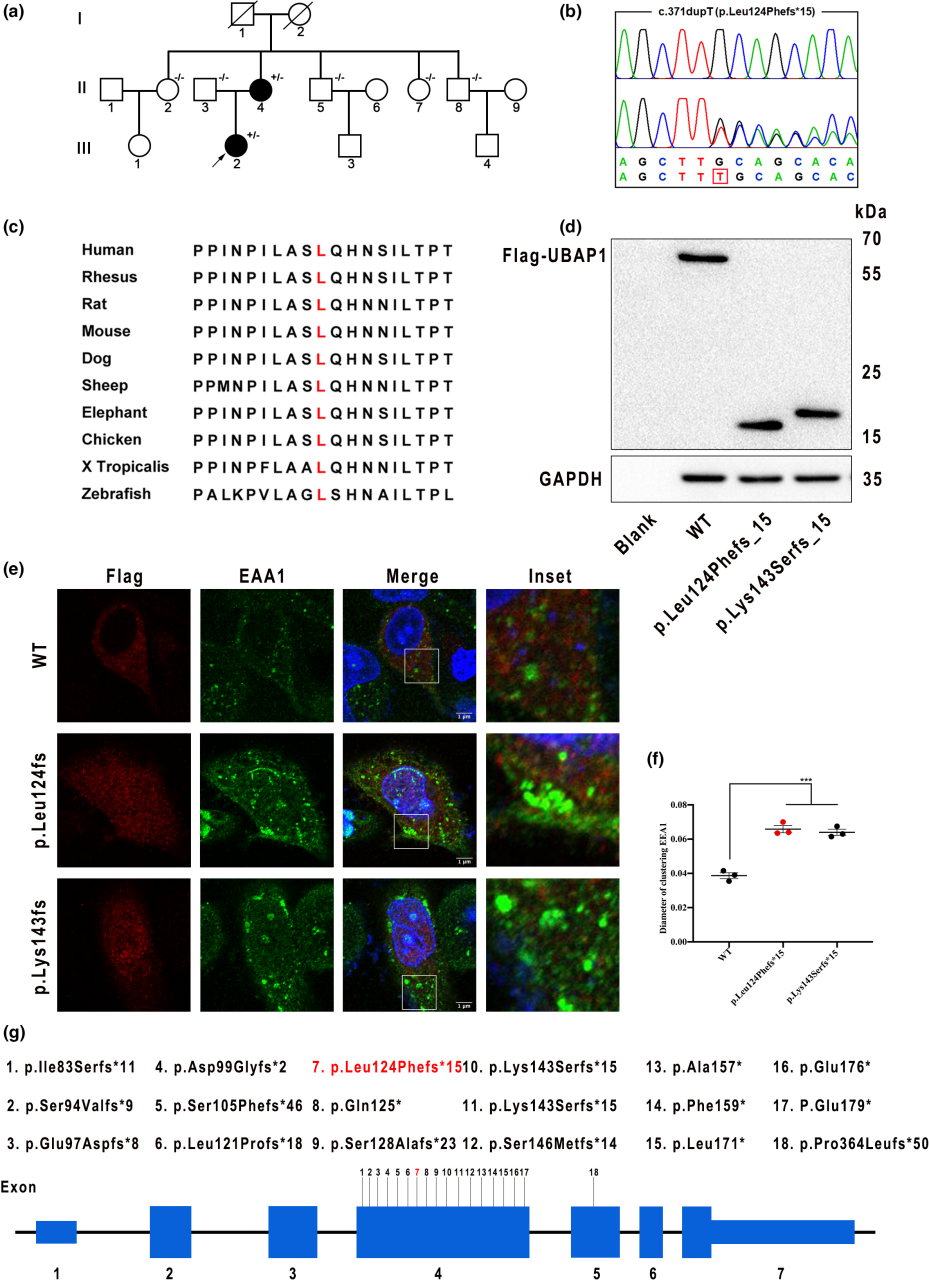
The p.Leu124Phefs*15 variant within *UBAP1* in a Chinese HSP family. (a) The pedigree shows segregation of the variant that has been confirmed. Circles denote females and squares denote males. “+/−” denotes the heterozygous variant in the *UBAP1* gene from individuals with HSP; “−/−” denotes no variant in the *UBAP1* gene from individual without HSP. (b) Sanger sequencing analysis traces. (c) Alignment analysis for the p.Leu124Phefs*15 variant within *UBAP1* orthologues in different species. Amino acid position 124 is in red. (d) HEK 293T cells were respectively transfected with WT and mutant UBAP1. Western blot analysis revealed that the variant (p.Leu124Phefs*15) actually led to the production of truncated mutant form of UBAP1 (p.Lys143Serfs*15 as positive control). (e) Flag‐tagged WT or mutant UBAP1 plasmids were transfected in HeLa cells, then visualized by anti‐flag (red) and anti‐EEA1 (green) immunofluorescence. Scale bar = 1 μm. (f) Measurement of the early endosome diameters by ImageJ (data are presented as means ± SEM of three experiments). (g) Schematic diagram of the locus of 18 variants within *UBAP1*. Known variants are shown in black and the novel one in red. Amino acid changes are predicted from transcript NM_016525.5

The proband is 33‐year‐old female with a history of progressive weakness and rigidity of lower limbs for 25 years. She had difficulty in climbing the stairs and walking stably. There is no muscle atrophy of lower extremities. Her mother had similar symptoms, while other familial members were unaffected. Physical examinations revealed normal muscle strength but increased muscle tension in lower extremities. Tendon reflex was brisk in four limbs and Babinski sign was positive bilaterally. Vibratory sensibility was lost in the distal end of lower limbs. Brain MRI revealed unremarkable information, except for several lacunar infarcts. Thoracic MRI displayed extensive atrophy. EMG revealed normal amplitude and conduction velocity of motor nerves and sensory nerves. We performed whole exome sequencing in the proband. After verifying by Sanger sequencing, we identified a heterozygous *UBAP1* truncated variant c.371dupT (p.Leu124Phefs*15) (Figure 1b). We then performed Sanger sequencing in her available family members and found that her affected mother carried the same c.371dupT variant. This variant was absent in the ExAC, 1000G, gnomAD, and our in‐house WES database that contain 500 Chinese controls. The affected residue was much conserved in different species (Figure 1c). According to the ACMG guideline (Richards et al., 2015), this variant should be assigned as pathogenic.

*UBAP1*, encodes the ubiquitin‐associated protein 1 (UBAP1), a subunit of ESCRT‐I complex. UBAP1 has two main domains, the UMA domain in the N‐terminal region (17–63 aa) and a SOUBA domain in the C‐terminal region. The former domain is associated with ESCRT‐I complex, while the latter domain maintains ubiquitin homeostasis of early endosome processing. We constructed plasmids containing wild‐type (WT) or mutant *UBAP1* gene (NM_016525.5) and transfected the plasmids in HEK 293T cells. Western blot analysis revealed that this truncating variant actually led to the production of truncated mutant form of UBAP1, lacking the SOUBA domain (Figure 1d). We performed immunocytochemical staining for the EEA1 (early endosome marker) and Flag‐fused UBAP1 in HeLa cells to elucidate the endosome function. The results showed the aberrant endosome aggregates (Figure 1e) and prominent enlarged endosome in cells over‐expressing mutant UBAP1 (Figure 1f).

To date, 18 *UBAP1* variants including the one identified here have been described (Bian et al., 2021; Bourinaris et al., 2020; Gu et al., 2020; Wang et al., 2020), and 17 of them occurred in Exon 4 of *UBAP1* (Figure 1g), implying that Exon 4 is a potential hotspot region of *UBAP1*. In addition, all identified variants preserve the UMA domain but cause a loss of the SOUBA domain, implying that loss of ubiquitin binding would be detrimental. Further studies are required to elucidate the mechanism of SOUBA impairment causing HSP.

In summary, we identified a novel *UBAP1* truncating variant in a Chinese autosomal dominant HSP pedigree. Our findings expanded variant spectrum of *UBAP1* and further confirmed the pathogenicity of *UBAP1* variants in HSP.

## CONFLICT OF INTEREST

The authors have declared no conflict of interest.

## AUTHOR CONTRIBUTIONS

Qiao Wei: data acquisition, analysis, and interpretation of data, statistical analysis, drafting the manuscript. Pei‐Shan Wang: data acquisition, analysis, and interpretation of data. Hai‐Lin Dong: data acquisition, interpretation of data. Wen‐Jiao Luo: data acquisition. Zhi‐Ying Wu and Hong‐Fu Li: funding, study design and conceptualization, data acquisition, analysis and interpretation of data, technical and material support, drafting, and critical revision of the manuscript.

## ETHICAL STATEMENT

The study was approved by the Ethics Committees of Second Affiliated Hospital of Zhejiang University School of Medicine and have therefore been performed in accordance with the ethical standards laid down in the 1964 Declaration of Helsinki and its later amendments. Specific national laws have been observed, too. Written informed consent was obtained for this study from all the patients prior to their inclusion in the study.

## Data Availability

Data available on request from the authors.
